# Berberine Moderates Glucose and Lipid Metabolism through Multipathway Mechanism

**DOI:** 10.1155/2011/924851

**Published:** 2010-09-26

**Authors:** Qian Zhang, Xinhua Xiao, Kai Feng, Tong Wang, Wenhui Li, Tao Yuan, Xiaofang Sun, Qi Sun, Hongding Xiang, Heng Wang

**Affiliations:** Department of Endocrinology, Peking Union Medical College Hospital, Chinese Academy of Medical Sciences, Beijing 100730, China

## Abstract

Berberine is known to improve glucose and lipid metabolism disorders, but the mechanism is still under investigation. In this paper, we explored the effects of berberine on the weight, glucose levels, lipid metabolism, and serum insulin of KKAy mice and investigated its possible glucose and lipid-regulating mechanism. We randomly divided KKAy mice into two groups: berberine group (treated with 250 mg/kg/d berberine) and control group. Fasting blood glucose (FBG), weight, total cholesterol (TC), triglyceride (TG), high-density lipoprotein-cholesterol (HDL-c), low-density lipoprotein-cholesterol (LDL-c), and fasting serum insulin were measured in both groups. The oral glucose tolerance test (OGTT) was performed. RT^2^ PCR array gene expression analysis was performed using skeletal muscle of KKAy mice. Our data demonstrated that berberine significantly decreased FBG, area under the curve (AUC), fasting serum insulin (FINS), homeostasis model assessment insulin resistance (HOMA-IR) index, TC, and TG, compared with those of control group. RT^2^ profiler PCR array analysis showed that berberine upregulated the expression of glucose transporter 4 (GLUT4), mitogen-activated protein kinase 14 (MAPK14), MAPK8(c-jun N-terminal kinase, JNK), peroxisome proliferator-activated receptor *α* (PPAR*α*), uncoupling protein 2 (UCP2), and hepatic nuclear factor 4*α*(HNF4*α*), whereas it downregulated the expression of PPAR*γ*, CCAAT/enhancer-binding protein (CEBP), PPAR*γ* coactivator 1*α*(PGC 1*α*), and resistin. These results suggest that berberine moderates glucose and lipid metabolism through a multipathway mechanism that includes AMP-activated protein kinase-(AMPK-) p38 MAPK-GLUT4, JNK pathway, and PPAR*α* pathway.

## 1. Introduction

Type 2 diabetes mellitus (T2DM) is a metabolic disorder characterized by dysregulation of carbohydrate, protein, and fat metabolism resulting from defects in insulin secretion, insulin action, or both [1]. The number of T2DM patients is expected to rise to 300 million worldwide by the year 2025 due to an increased number of elderly people, a greater prevalence of obesity, and sedentary lifestyles [2]. Besides hyperglycemia, several other symptoms, including hyperlipidemia, are involved in the development of microvascular and macrovascular complications of diabetes, which are the major causes of morbidity and death. Therefore, it is especially important to reinforce effective prevention and regular treatment of this disease. However, since patient compliance with diet and exercise regiments is often poor and medications are needed and because many oral medications have a number of serious adverse effects, management of hyperglycemia or hyperlipidemia with low side effects remains a challenge to the medical system. Traditional Chinese medicines and their extractions demonstrate the characteristics of economy and effectiveness in managing diabetes and its complications.

*Rhizoma Coptidis* was recorded as an antidiabetes medication about 1500 years ago in a book titled “Note of Elite Physicians” by Hongjing Tao. Berberine is the major active component of *Rhizoma coptidis*. Recent studies have demonstrated beneficial effects of berberine on metabolism disorders including weight control, cholesterol reduction, antilipogenic and hypoglycemic effects, and even inhibiting chronic cocanine-induced sensitization [3–6].

Many studies have been published on the glucose-reducing mechanism of berberine. Zhou et al. found that berberine stimulated glucose transport through a mechanism distinct from insulin in 3T3-L1 adipocyes [7]. Moreover, berberine could activate AMPK and induced glycolysis in L6, C2C12, and 3T3-L1 cell lines [8]. And berberine dose-dependently inhibited respiration in L6 myotubes by its specific effect on respiratory complex I [9]. Regarding the mechanism of berberine in moderating lipid metabolism, Lee et al. found that berberine moderated lipids by inhibiting adipogenesis in 3T3-L1 adipocytes [10]. Two trials revealed that berberine activated extracellular signal-regulated kinase (ERK) [7] and JNK [11] in HepG2 cells. Given these results, we hypothesize that berberine may exhibit a multitargeted mechanism in moderating glucose and lipids.

Trial materials used in biomedical studies often involve cells. However, in this study, KKAy mice were used to investigate the effects of berberine on glucose and lipid metabolism in vivo. KKAy mice are developed by transferring the yellow obesity (Ay) gene into the KK strain, which show severe obesity, hyperglycemia, hyperinsulinemia, and glucose intolerance by eight weeks of age. So, they are especially useful for evaluating of antidiabetic and antiobesity agents. The skeletal muscle plays a major role in energy balance. It accounts for >30% of energy expenditure and is the primary tissue of insulin stimulating glucose uptake, disposal, and storage [12]. To understand the mechanism that berberine regulates glucose and lipids, we performed RT^2^ PCR diabetes superarray to analyze the expression of diabetes-related genes in skeletal muscle tissue of KKAy mice. Natural products are gaining increased applications in drug discovery and development. Being chemically diverse, they are able to modulate several targets simultaneously in a complex system. DNA microarrays serve as suitable high-throughput tool for simultaneous analysis of multiple genes [13].

## 2. Materials and Methods

### 2.1. Animal Modeling, Grouping, and Treatment

Male KKAy mice (from the Chinese Academy of Medical Sciences, Beijing, China) were fed in the standard mouse-feeding room. The mice were fed with high-fat laboratory chow (fat: carbohydrate: protein = 58 : 25.6 : 16.4). All procedures were approved by the Ethics Committee for the Use of Experimental Animals of Peking Union Medical College Hospital. Before drug administration, murine blood samples for blood glucose measurement were collected from the tail vein. KKAy mice with random blood glucose values above 11.1 mmol/L were considered diabetic. These mice were randomly divided into two groups: berberine group (*n* = 8, ig 250 mg*·*kg^−1^ *·*d^−1^ berberine) and control group (*n* = 8, ig the same volume of normal saline). Drugs were given to the mice between 8:00 and 9:00 AM every day. Over a four-week period, on days 0 (before treatment), 7, 14, 21, and 28, weight and FBG of the KKAy mice (4-hour fast) were measured in blood samples obtained from tail veins. An oral glucose tolerance test (OGTT) was performed on day 21. On day 28, blood samples of the KKAy mice were again taken for measuring FINS and lipid metabolic parameters, after anesthesia. The mice were then sacrificed and their skeletal muscles were collected and stored in dry ice.

### 2.2. Oral Glucose Tolerance Test (OGTT)

After the mice fasted for 4 hours, glucose 2.2 g/kg was orally administered. Then blood samples were collected from tail veins at 0 (prior to glucose load), 30, 60, and 120 minutes (after glucose load) for the glucose assay. AUC was calculated for blood glucose (BG) during the OGTT: AUC = 0.5 × [Bg0 + Bg30]/2 + 0.5 × [Bg30 + Bg60]/2 + 1 × [Bg60 + Bg120]/2.

### 2.3. Measurement of Serum Parameters

Blood glucose was measured by the glucose oxidase peroxidase (Bayer Breeze blood glucose meter, Germany) method. TC, TG, LDL-c, and HDL-c were assayed by enzyme end-point method (Roche, Germany). Serum insulin was measured by enzyme-linked immunosorbent assay (ELISA) using rat/mice insulin ELISA kit (LINCO Research, USA). HOMA-IR index was calculated according to the following formula: HOMA-IR = FBG (mmol/L) × FINS (*μ*U/mL)/22.5.

### 2.4. RT^2^ Profiler PCR Array

#### 2.4.1. First Strand cDNA Synthesis

Total RNA was extracted from the skeletal muscle of 3 mice from the berberine group and 3 mice from the control group, using TRIZOL Reagent (Invitrogen Life Technologies, USA). RNA cleanup used RNeasy MinElute Cleanup Kit (Qiagen, Germany). RNA quality was determined by running a sample with RNA loading dye (Ambion, USA) on a 1% agarose gel and inspecting for distinct 18S and 28S bands, indicating lack of degradation. Quantity was determined by A260 and A280 measurement. All samples had A260/A280 ratios of 1.9–2.1. SuperScript III Reverse Transcriptase was applied to reverse-transcribe RNA into first-strand cDNA. To analyze the differential expression of multiple genes involved in diabetes mellitus, we used RT^2^ profiler PCR mouse diabetes mellitus-specific expression arrays (SuperArray, Frederick, MA, USA), which uses SYBR Green-based real-time PCR to assay a large number of genes simultaneously. Each superarray membrane contained 84 specific cDNA fragments of genes involved in diabetes mellitus, including receptors, transporters and channels, nuclear receptors, metabolic enzymes, secreted factors, transcription factors, and others. Table 1lists the genes measured in this study. We added cDNA to each well of an RT^2^ profiler PCR diabetes array for quantitative PCR in the ABI PRISM 7700 system (Applied Biosystems, USA) with the following cycling conditions: an initial denaturation at 95°C 15 minutes, and 40 cycles of 95°C 15 seconds, 55°C 60 seconds, with a final infinite 4°C hold. Fluorescence signal was then collected. For quality control purpose, no reverse transcription control and no template control were performed.

#### 2.4.2. Data Normalization and Analysis

Five endogenous control genes—glucuronidase *β*(GUS *β*), hypoxanthine guanine (HPRT1), heat-shock protein (HSP90), glyceralsehyde(GAPDH), and *β*-actin(ACT *β*)—present on the PCR Array were used for normalization. Each replicate cycle threshold (Ct) was normalized to the average Ct of 5 endogenous controls on a per plate basis. The comparative Ct method was used to calculate the relative quantification of gene expression. The following formula was used to calculate the relative amount of the transcripts in the berberine-treated samples and samples of the control group, both of which were normalized to the endogenous controls. ΔΔCt = ΔCt (berberine group)—ΔCt (control group) for RNA samples [14]. ΔCt is the log  2 difference in Ct between the target gene and endogenous controls abstained by subtracting the average Ct of controls from each replicate. The fold change for each berberine-treated sample relative to the control sample = 2^−ΔΔCt^.

#### 2.4.3. Sensitivity Detection and Identification Expressed Genes

PCR Array quantification was based on the Ct number. Ct was defined as 35 for the ΔCt calculation when the signal was under detectable limits. A list of differentially expressed genes was identified using a 2-tailed *t*-test. Changes in gene expression between the berberine group and the control group were illustrated as a fold increase/decrease. The criteria were a *P* value <.05 and a mean difference equal to or greater than 2-fold. The statistical calculation was based on the web-based program of RT^2^ profiler PCR Array Data Analysis. Genes that suited the above criteria were considered to be up- or downregulated. The experiments were repeated three times.

### 2.5. Statistical Analysis

All results are expressed as means ± SD. The statistical value of *P* < .05 was considered as statistical significance. Differences between the berberine group and the control group were determined using the *Mann-Whitey U* test. All statistical analyses were performed with SPSS version 12.0 software.

## 3. Results

### 3.1. Berberine Showed no Effect on Body Weight of KKAy Mice

**Figure 1:**
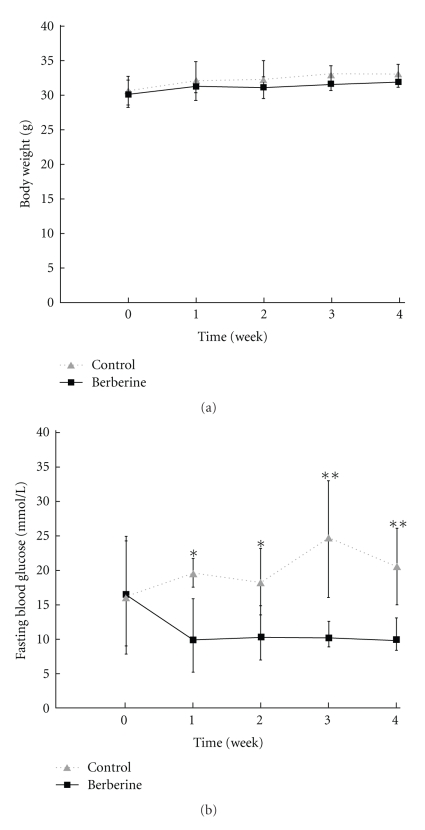
Weekly variation of body weight (a) and fasting blood glucose (b) between berberine group and control group. Data are expressed as means ± SD. *P < .05, **P < .01 compared with that in the control.

No significant differences were noted between the initial body weight of KKAy mice in the berberine group and the control group. Also, after treatment, no significant differences were found between groups, suggesting that berberine does not affect body weight (Table 2, Figure 1(a)).

### 3.2. Berberine Decreased FBG of KKAy Mice

Before drug administration, FBG in both groups was similar. However, the FBG in the berberine group displayed a significant decrease on day 7 (*P* < .05), day 14 (*P* < .05), day 21 (*P* < .01), and day 28 (*P* < .01) (Table 2, Figure 1(b)). This showed that administration of berberine reduced the FBG of KKAy mice.

### 3.3. Berberine Improved the Glucose Tolerance of KKAy Mice

**Figure 2:**
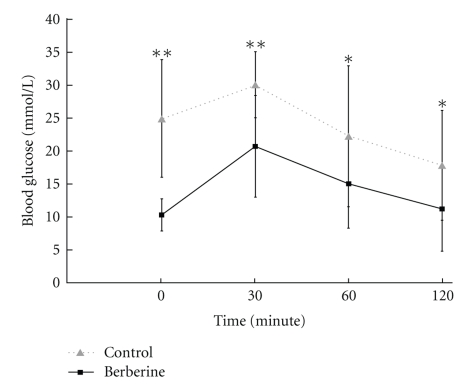
Comparison of plasma glucose concentration responses to an oral glucose tolerance test between berberine group and control group. Data are expressed as means ± SD. *P < .05, **P < .01 compared with that in the control.

**Figure 3:**
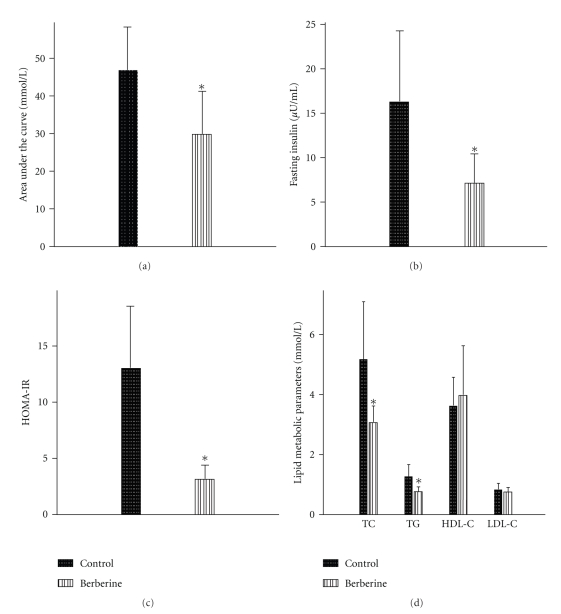
Area under the curve in OGTT trial (a), serum concentration of fasting insulin (b), HOMA-IR, (c) and lipid metabolic parameters (d) between berberine group and control group. Data are expressed as means ± SD. *P < .05 compared with that in the control.

Blood glucose levels in two groups became elevated within 30 minutes after the administration of glucose to mice. Blood glucose levels recovered to the original levels after 120 minutes. The blood glucose level in mice treated with berberine was significantly suppressed at 0 minutes (*P* < .01), 30 minutes (*P* < .01), 60 minutes (*P* < .05), and 120 minutes (*P* < .05) compared to those in the control group (Table 3, Figure 2). AUC in the berberine group was significantly smaller than that in the control group (*P* < .05) (Table 3, Figure 3(a)). This indicated that berberine improved the glucose tolerance of KKAy mice.

### 3.4. Berberine Reduced FINS and HOMA-IR in KKAy Mice

The mice fed with berberine exhibited significantly lower fasting serum insulin levels than the control group (*P* < .05) (Table 4, Figure 3(b)).The HOMA-IR index of the berberine group was 30% of that of the control group (*P* < .05) (Table 4, Figure 3(c)).These data showed that berberine ameliorated the insulin sensitivity of KKAy mice.

### 3.5. Berberine Declined TC and TG in KKAy Mice

In addition to its glucose-lowering effects, berberine may have lipid-regulating activity. After 4 weeks of treatment, the animals fed with berberine exhibited a significant decrease in TC (*P* < .05) and TG level (*P* < .05). However, differences in HDL-c and LDL-c between the two groups were not statistically significant (Table 4, Figure 3(d)).

### 3.6. RT^2^ Profiler PCR Array

Based on the results of the RT^2^ profiler PCR array, we found no significantly different genes expression between berberine and control group related to the insulin signaling pathway. For example, gene expression of thymoma viral proto-oncogene 2(AKT2), inhibitor of kappaB kinase *β* (IKBKB), insulin receptor substrate (IRS-1), phosphatidlyinositol-3 kinase regulatory subunit polypeptide 1 (PIK3R1), phosphatidylinositol 3-kinase catalytic *δ* polypeptide (PIK3CD), and protein tyrosine phosphatase nonreceptor type 1(PTPN1,PTP1B), all of which are involved in the insulin signaling pathway, were not markedly upregulated or downregulated.

**Figure 4:**
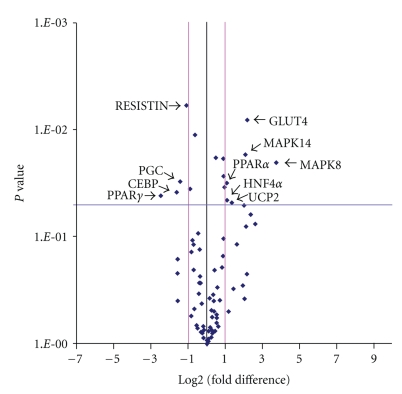
The Volcano Plot graphs of superarray. This graph shows that the log  2 of the fold change in each gene's expression between berberine group and control group is versus its *P* value from the *t*-test. The black line indicates fold changes of 1. The pink lines indicate that the fold change in gene expression threshold is 2. The blue line indicates that the *P* value of the *t*-test threshold is  .05. There were 10 genes which showed significantly different expression between berberine group and control group.

Based on gene selection criteria (*P* < .05 and fold change ≥2), 10 genes that were ≥2-fold upregulated or downregulated in the berberine group as shown in Table 5 and Figure 4.

According to the gene expression profile, we found that berberine could upregulated the expression of 2 genes related to the MAPK (mitogen-activated protein kinase) pathway. For example, gene expression of MAPK8 and MAPK14, both of which are involved in the MAPK pathway, was upregulated (13.15-fold and 4.17-fold, resp.) in the berberine group. Also, glucose transporter 4 (GLUT4) gene expression, which is involved in glucose transport in membranes, was upregulated by 4.48-fold in the berberine group. Gene expression of peroxisome proliferator-activated receptor *γ* (PPAR*γ*), CCAAT/enhancer-binding protein (CEBP), and PPAR*γ* coactivator 1*α* (PGC), which play important roles in adipogenesis, were downregulated (5.51-fold, 3.06-fold, and 2.70-fold, resp.). PPAR*α* gene expression, which leads to reduction of plasma TG levels, was upregulated by 2.10-fold in the berberine group. In addition, gene expression of uncoupling protein 2 (UCP2), whose expression is involved in energy balance, was upregulated by 2.55-fold in the berberine group. Furthermore, expression of hepatic nuclear factor 4*α*(HNF-4*α*) gene, a key regulator of glucose, cholesterol, and fatty acid metabolism, was upregulated by 2.12-fold in the berberine group. Gene expression of resistin, which contributes to insulin resistance, was downregulated by 2.12-fold in the berberine group.

## 4. Discussion

In this study, we found that the administration of berberine to KKAy mice significantly reduced FBG levels, FINS, and HOMA-IR index. Our results suggest that berberine has glucose-moderating action and can ameliorate oral glucose tolerance and insulin sensitivity. This result is similar to the results of other studies [7, 8, 10, 15–17]. However, Lee et al. treated ob/ob mice with berberine for 25 days and found that berberine could reduce the body weight [10]. In our study, berberine did not reduce body weight significantly. Othermore, berberine decreased serum TG and TC levels in KKAy mice. Two groups revealed the similar results [18, 19]. However, Kong et al. found that oral administration of berberine in 32 hypercholesterolemic patients for 3 months reduced LDL-cholesterol by 25% [19]. While in this study, we found no evidence that berberine reduces serum LDL-c.

The mechanism of the hypoglycemic action of berberine is still under investigation. Our gene expression profile results showed no significant differences in gene expression between the berberine and the control group related to the insulin signaling pathway, including AKT2, IKBKB, IRS-1, PIK3R1, PIK3CD, and PTPN1 (PTP1B). In the insulin signaling pathway, insulin stimulates glucose uptake to muscle and fat tissue [20]. It involves certain signal molecules, such as insulin receptor (IR), IRS-1, phosphatidylinositol 3-kinase (PI3K), and AKT. Defects are found in the insulin signaling pathway in muscle tissue of T2DM. Based on our results, we do not consider the insulin signaling pathway to be the key mechanism of the glucose-moderating effect of berberine. Other research supports this view. Berberine only weakly stimulated the phosphorylation of AKT/PKB [21] and did not augment tyrosine phosphorylation of IR and IRS-1 in 3T3-L1 cells [22]. Unlike insulin, the effect of berberine on increasing glucose uptake was insensitive to wortmannin, an inhibitor of PI3K, and SB203580, an inhibitor of p38 MAPK [7]. IKBKB can phosphorylate IRS1 on Ser307, thereby rendering IRS1 less amenable to become phosphorylated on tyrosine residues required to propagate the signal to downstream effectors such as protein kinase B [23]. PTP1B may dephosphorylate IRS-1 [24, 25]. Other studies suggest that PTP1B polymorphisms may be associated with obesity and insulin resistance in humans [26–28]. The overexpression of PTP1B in muscle tissue results in insulin resistance [29].

We disclosed that berberine could upregulate the expression of 2 genes related to MAPK (mitogen-activated protein kinase) pathway and GLUT4. This suggests that berberine can activates MAPK in KKAy mice. The p38 MAPK cascade is downstream of AMPK in the signaling pathway of AICAR-stimulated glucose transport in Clone 9 cells [30, 31]. In muscle tissue, p38 MAPK takes part in the activation of GLUT4 [30, 32]. It is possible that berberine can ameliorate glucose uptake in T2DM mice through the AMPK-p38 MAPK-GLUT4 pathway. The possibility that berberine activate MAPK is still under investigation. Cheng et al. found that berberine strongly promotes the phosphorylation of AMPK and p38 MAPK on L6 rat skeletal muscles [21]. Moreover, berberine-stimulated glucose uptake was inhibited by the AMPK inhibitor Compound C and the p38 MAPK inhibitor SB202190. Inhibition of AMPK reduced p38 MAPK phosphorylation, suggesting that AMPK lies upstream of p38 MAPK. These results suggest that berberine circumvents insulin signaling pathways and stimulates glucose uptake through the AMP-AMPK-p38 MAPK pathway, which may account for the antihyperglycemic effects of this drug [21]. Zhou et al. found that in isolated rat skeletal muscles, metformin stimulates glucose uptake coincident with AMPK activation [33]. However, Lee et al. revealed that berberine increased AMPK phosphorylation, but it did not activate p38 [10]. Several groups investigated the effect of berberine on glucose transporters and their conclusions remain controversial. Two groups also reported that berberine was able to stimulate GLUT4 translocation [10, 34], but this activity was not observed by other groups [10, 22]. Repeated intravenous administration of tetrandrine (1.0 mg/kg) to STZ-diabetic rats for 3 days resulted in an increase in the mRNA and protein levels of the GLUT4 in soleus muscle, in addition to the lowering of plasma glucose [35].

Although, the exact mechanisms of berberine lipid-modulating effects are still unknown, we found that berberine could downregulate PPAR*γ*, PGC-1, and C/EBP*α* and upregulate PPAR*α*. The nuclear receptor PPAR*γ* and members of the C/EBP family take important roles in adipogenesis [36]. The expression of both gluconeogenic and *β*-oxidation genes is further potentiated by the nuclear hormone receptor coactivator PGC-1, a target of cyclic AMP (cAMP) response element binding (CREB), whose levels are increased during fasting and in diabetes [37, 38]. Berberine could downregulate the expression of PPAR*γ*, PGC-1, and C/EBP levels, which may be the mechanism that prevented KKAy mice treated with berberine from gaining weight. Weight gain has been identified as a class effect of the thiazolidinediones (TZDs). A factor contributing to TZD-related weight gain is increased adipocyte differentiation. The antidiabetic effects of TZDs are mediated through PPAR*γ*, a positive regulator of adipocyte differentiation [39]. Activation of PPAR*γ* triggers the production of smaller more insulin-sensitive adipocytes, predominantly in the subcutaneous adipose compartment, and is likely to contribute to the TZD-mediated weight gain that has been observed in both animal and human studies [40]. Obesity is a major risk factor for metabolic syndrome and T2DM. However, most antidiabetic drugs that are hypoglycemic also promote weight gain, alleviating one symptom of T2DM while aggravating a major risk factor that leads to T2DM. It is highly desirable to develop pharmaceuticals and treatments for T2DM that reduce blood glucose levels without inducing adipogenesis in patients. Many studies have showed that berberine inhibited the mRNA and protein levels of adipogenesis-related transcription factors PPAR*γ* and C/EBP*α* [10, 41, 42]. Two groups confirmed that berberine increased PPAR*α*/*δ* expression in liver and skeletal muscle tissue of diabetic rats [43, 44]. PPAR*α* governs the expression of numerous genes involved in a variety of metabolic processes and modulates lipoprotein metabolism in several ways. Activation of PPAR*α* results in a reduction of plasma TG levels, which is achieved by induction of genes that decrease the availability of TG for hepatic VLDL secretion and induction of genes that promote lipoprotein lipase-mediated lipolysis of TG-rich plasma lipoproteins [45]. Upregulation of PPAR*α* may be the mechanism of the action of berberine in moderating lipid metabolism, just as in fibrate.

We also found that berberine could upregulate MAPK8 (JNK1). Berberine could increase the transcriptional activity of LDLR promoter and it involved the JNK pathway, reducing serum LDL [11]. Oral administration of berberine in 32 hypercholesterolemic patients for 3 months reduced serum cholesterol by 29%, triglycerides by 35%, and LDL-cholesterol by 25% [19]. Treatment of hyperlipidemic hamsters with berberine reduced serum cholesterol by 40% and LDL-cholesterol by 42%, with a 3.5-fold increase in hepatic LDLR mRNA, and a 2.6-fold increase in hepatic LDLR protein [19]. Using human hepatoma cells, berberine upregulated LDLR expression independent of sterol regulatory element binding proteins but was dependent on ERK activation [46]. Although we found no evidence that berberine can reduce serum LDL, the upregulation of MAPK is a possible factor in the berberine lipid-regulating mechanism.

Moreover, berberine could upregulate UCP2. UCP2, which is found in many tissues, affects body weight gain, resting metabolic rates and food intake, which are all involved in energy balance [47]. In adipocytes and skeletal muscle tissue, UCP2 expression appears to increase following high-fat feeding. An increase in UCP2 levels might be expected to increase energy expenditure and decrease the risk of obesity. In skeletal muscle, the expression of UCP2 mRNA was significantly increased by berberine [10]. We speculated that bererine could upregulate UCP2, increase energy dissipation, and decrease the risk of obesity.

Also, we revealed that berberine could downregulate resistin. Resistin, as an adipocyte-secreted factor (ADSF), is a mouse protein with potential roles in insulin resistance and adipocyte differentiation. The TZD drug rosiglitazone can reduce resistin expression to improve insulin resistance. Ding et al. found that the effects of berberine in moderating lipids, as well as its contribution to reducing leptin and resistin, were closely correlated with its effects in ameliorating insulin resistance [48].

**Figure 5:**
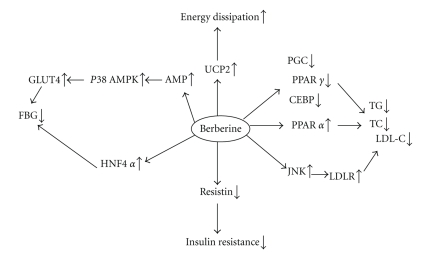
The mechanism of berberine moderating glucose and lipid metabolism.

Additionally, berberine upregulated hepatic nuclear factor 4*α*(HNF4*α*). HNF4*α* is a key regulator of a number of genes involved in glucose, cholesterol, and fatty acid metabolism [49]. The mRNA and protein expression of HNF-4*α* were decreased in fructose-fed rats, but berberine promoted its expression [50].  Figure 5 shows the mechanism of berberine in moderating glucose and lipid metabolism.

In summary, our studies provide evidence that berberine reduces FBG and FINS and ameliorates insulin sensitivity and secretion in KKAy mice. Moreover, berberine reduces serum TC and TG and thereby regulates lipid metabolism. Berberine, in our study and others, caused changes in the expression of many genes that are involved in AMPK-p38 MAPK-GLUT4, JNK pathway, and PPAR*α* pathway. These results provide molecular information for further investigation of the mechanisms by which berberine moderates glucose and lipid metabolism. Furthermore, these results could be important in devising mechanism-based and targeted therapeutic strategies for diabetes and lipid disorders. Further studies (i.e., real-time quantitative PCR) are planned to validate the gene expression profile changes observed with the RT^2^ Profiler PCR array.

## Figures and Tables

**Table 1:** Gene list of RT^2^ profiler PCR mouse diabetes array.

Ace	Acly	Adra1a	Adrb3	Agt	Akt2	Aqp2	Ccl5	Ccr2	Cd28	Ceacam1	Cebpa
Ctla4	Dpp4	Dusp4	Enpp1	Fbp1	Foxc2	Foxg1	Foxp3	G6pc	G6pd2	Gcg	Gcgr
Glp1r	Gpd1	Gsk3b	Hmox1	Hnf4a	Icam1	Ide	Ifng	Igfbp5	Ikbkb	Il10	Il12b
Il4ra	Il6	Inppl1	Ins1	Pdx1	Irs1	Mapk14	Mapk8	Neurod1	Nfkb1	Nos3	Nrf1
Nsf	Parp1	Pax4	Pck1	Pfkfb3	Pik3cd	Pik3r1	Ppara	Pparg	Ppargc1a	Ptpn1	Pygl
Rab4a	Retn	Sell	Serpine1	Slc14a2	Slc2a4	Snap23	Snap25	Sod2	Srebf1	Stx4a	Stxbp1
Stxbp4	Hnf1b	Tgfb1	Tnf	Tnfrsf1a	Tnfrsf1b	Trib3	Ucp2	Vamp2	Vamp3	Vapa	Vegfa
Gusb	Hprt1	Hsp90ab1	Gapdh	Actb	MGDC	RTC	RTC	RTC	PPC	PPC	PPC

**Table 2:** Effect of berberine on the body weight and FBG of KKAy mice.

Group	*n*	Day 0	Day 7	Day 14	Day 21	Day 28
*Control*						
Weight (g)	8	30.51 ± 2.44	31.92 ± 3.28	32.21 ± 3.15	32.90 ± 1.34	33.04 ± 1.42
FBG (mmol/L)	8	16.15 ± 8.98	19.60 ± 2.15	18.28 ± 5.05	24.74 ± 8.89	20.54 ± 5.85
*Berberine*						
Weight (g)	8	30.08 ± 1.94	30.86 ± 1.08	31.04 ± 1.76	31.55 ± 1.17	31.88 ± 0.74
FBG (mmol/L)	8	16.45 ± 8.04	9.90 ± 5.69*	10.35 ± 4.14*	10.20 ± 2.48**	9.90 ± 2.95**

Values are means ± SD.

*Indicates significantly different versus control (*P* < .05); **(*P* < .01).

**Table 3:** Effect of berberine on oral glucose tolerance in KKAy mice (mmol·L^−1^).

Group	*n*	BG 0 min	BG 30 min	BG 60 min	BG 120 min	AUC
Control	8	24.74 ± 8.89	29.90 ± 4.98	22.10 ± 10.67	17.70 ± 8.29	45.45 ± 10.81
Berberine	8	10.20 ± 2.48**	20.58 ± 7.69**	14.90 ± 6.69*	11.08 ± 6.36*	29.90 ± 11.89*

Values are means ± SD.

*Indicates significantly different versus control (*P* < .05); **(*P* < .01).

**Table 4:** Effect of berberine on FINS, HOMA-IR, and lipid metabolic parameters in KKAy mice.

Group	*n*	FINS (*μ*U/mL)	HOMA-IR	TC (mmol/L)	TG (mmol/L)	HDL-c (mmol/L)	LDL-c (mmol/L)
Control	8	17.15 ± 7.74	13.45 ± 5.07	5.20 ± 1.93	1.29 ± 0.40	3.66 ± 0.95	0.86 ± 0.20
Berberine	8	6.80 ± 3.36*	3.05 ± 1.45*	3.03 ± 0.69*	0.75 ± 0.25*	4.00 ± 1.12	0.80 ± 0.12

Values are means ± SD.

*Indicates significantly different versus control (*P* < 0.05).

**Table 5:** The genes that expressed differently between berberine group and control group (fold change ⩾2.0, *P* < .05).

Gene name	Description	GeneBank ID	Fold change	*P* value *t*-test
SLC2A4	Solute carrier family 2(GLUT4)	NM_009204	4.48	.0081
MAPK8	Mitogen-activated protein kinase 8(JNK)	NM_016700	13.15	.0203
MAPK14	Mitogen-activated protein kinase 14	NM_011951	4.17	.0171
PPAR*α*	Peroxisome proliferator-activated receptor alpha	NM_011144	2.10	.0316
PPAR*γ*	Peroxisome proliferator-activated receptor gamma	NM_011146	−5.51	.0412
CEBP	CCAAT/enhancer-binding protein, alpha	NM_011144	−3.06	.0385
PPAR*γ*C1*α*	Peroxisome proliferative-activated receptor, gamma, coactivator 1 alpha(PGC)	NM_008904	−2.70	.0306
UCP2	Uncoupling protein 2	NM_011671	2.55	.0480
RETN	RESISTIN	NM_022984	−2.12	.0059
HNF4*α*	Hepatic nuclear factor 4, alpha	NM_008261	2.12	.0455
